# High-technology telerehabilitation for the treatment of frail and pre-frail patients: the TeleRiab4FRA study design and protocol

**DOI:** 10.3389/fdgth.2026.1929955

**Published:** 2026-08-24

**Authors:** Alessia Bramanti, Michele Ciccarelli, Albino Carrizzo, Placido Bramanti, Valeria Visco, Mariaconsiglia Calabrese, Marina Garofano, Maria Pia Di Palo, Colomba Pessolano, Simone Romano, Sabato Nocera, Giuseppe Scanniello, Carmine Vecchione

**Affiliations:** 1 Department of Medicine, Surgery and Dentistry, University of Salerno, Baronissi, Italy; 2 Vascular Pathophysiology Unit, IRCCS Neuromed, Isernia, Italy; 3 Faculty of Psychology, University ECampus, Novedrate, Italy; 4 Department of Computer Science, University of Salerno, Fisciano, Italy

**Keywords:** cardiac telerehabilitation, chronic heart failure, cognitive training, digital health, frailty, pre-frailty, remote monitoring, sensorimotor training

## Abstract

**Introduction:**

Chronic heart failure (CHF) in older adults is frequently associated with frailty, reduced functional capacity, cognitive vulnerability, poor quality of life, and recurrent healthcare needs. Despite the recognized benefits of cardiac rehabilitation, access and adherence to conventional center-based programs remain suboptimal in frail and pre-frail patients. The TeleRiab4FRA study investigates whether an 8-week high-technology telerehabilitation program combining sensorimotor and cognitive training can improve functional capacity and multidimensional outcomes in frail and pre-frail older adults with CHF.

**Methods:**

TeleRiab4FRA is a randomized, controlled, prospective, monocentric, parallel-group, open-label, non-profit clinical trial enrolling approximately 80 patients aged ≥65 years with stable CHF, New York Heart Association functional class I–III, and documented frailty or pre-frailty. Participants are randomized in a 1:1 ratio to a telerehabilitation group or to a caregiver-supervised home-based rehabilitation group. The telerehabilitation intervention is delivered through a synchronous digital platform using CE-marked devices for remote monitoring of electrocardiographic tracing, heart rate, peripheral oxygen saturation, and blood pressure. Both groups receive an 8-week individualized program including motor and cognitive training tailored according to frailty status. Assessments are performed at baseline, 4 weeks, 8 weeks, 16 weeks, and 24 weeks. The primary endpoint is the change in peak oxygen uptake (VO₂peak) measured by cardiopulmonary exercise testing from baseline to 8 weeks. Secondary outcomes include frailty status assessed by the Italian Frailty Index, physical performance, cognitive function, health-related quality of life, adherence, caregiver burden, perceived stress, technological usability, biomarkers, and safety events.

**Expected results:**

We hypothesize that high-technology telerehabilitation will lead to greater improvement in functional capacity than caregiver-supervised home-based rehabilitation. We further hypothesize favorable effects on frailty status, physical performance, cognitive outcomes, adherence, usability, and safety, with maintenance of any functional benefits at the 24-week follow-up. These statements represent prespecified hypotheses and not study findings.

**Conclusion:**

TeleRiab4FRA is designed to evaluate the clinical utility, feasibility, and safety of a synchronous, technology-enabled telerehabilitation model for frail and pre-frail older adults with CHF. If effective, this approach may support the development of scalable, personalized, and patient-centered rehabilitation pathways integrating clinical supervision, digital monitoring, and home-based care.

**Clinical trial registration:**

ClinicalTrials.gov, identifier NCT07561021.

## 1 Introduction

Heart failure (HF) is a major public health burden and one of the leading causes of morbidity, recurrent hospitalization, functional decline, and reduced quality of life among older adults (1, 2). In this population, the clinical complexity of HF is frequently amplified by frailty, a multidimensional condition characterized by reduced physiological reserve, decreased resilience to stressors, and increased vulnerability to adverse outcomes (3). Frailty and HF commonly coexist and may reinforce each other through a bidirectional relationship: HF can accelerate the progression of frailty by promoting exercise intolerance, neurohormonal activation, systemic inflammation, sarcopenia, fatigue, and reduced mobility, while frailty may worsen HF prognosis by increasing the risk of disability, hospitalization, dependency, poor adherence to treatment, and mortality (4).

Cardiac rehabilitation (CR) is a core component of comprehensive HF management. It has been shown to improve exercise capacity, symptoms, functional performance, and health-related quality of life (5). However, despite its recognized clinical value, participation in conventional center-based CR remains suboptimal, particularly among older frail and pre-frail patients (6, 7). Mobility limitations, transportation difficulties, geographical distance from specialized centers, fatigue, comorbidities, caregiver dependency, and the burden of repeated hospital visits may limit access, adherence, and continuity of care (8). These barriers highlight the need for alternative rehabilitation models that are safe, personalized, accessible, and capable of supporting long-term management in the home environment (9).

Telerehabilitation has emerged as a promising digital health strategy to overcome some of these limitations (10). By combining remote supervision, synchronous interaction with healthcare professionals, wearable or connected monitoring devices, sensor-based assessment, and structured home-based exercise programs, telerehabilitation may extend rehabilitation services beyond traditional clinical settings (11, 12). In patients with HF, technology-supported CR has shown encouraging results in terms of feasibility, adherence, functional capacity, and quality of life (11, 13). Nevertheless, evidence remains limited for frail and pre-frail older adults, who represent a clinically vulnerable population with specific functional, cognitive, safety, and caregiver-related needs (7, 14).

Randomized studies and evidence syntheses suggest that home-based telerehabilitation can improve access, exercise capacity, and selected patient-reported outcomes in HF; however, the available interventions remain heterogeneous. Earlier programs have often emphasized exercise training, telephone or video coaching, or physiological telemonitoring, with usual care or remote patient management as the comparator. Frail and pre-frail patients have frequently been underrepresented, cognitive training has rarely been integrated as a structured treatment component, and follow-up and real-time multimodal clinical monitoring have varied across studies (13–17). These limitations support the need for trials specifically designed around the functional, cognitive, safety, and caregiver-related needs of older adults with HF and frailty.

Frailty is not limited to physical impairment: cognitive decline, reduced executive function, slowed psychomotor performance, and lower self-management capacity may coexist with physical vulnerability and negatively affect participation in rehabilitation programs (18). For this reason, interventions designed for frail patients with HF should not be restricted to aerobic or resistance exercise alone, but should adopt a multidimensional approach integrating motor, cognitive, educational, and monitoring components (19, 20). A technology-enabled rehabilitation model may be particularly suitable when it allows real-time professional supervision, continuous monitoring of vital parameters, individualized progression of exercise intensity, and adaptation of the program according to the patient's clinical and functional profile (11, 21).

The biological rationale for combining motor and cognitive training is based on the partially shared mechanisms underlying HF, frailty, and cognitive vulnerability. Chronic low-grade inflammation, vascular and endothelial dysfunction, reduced cerebral and skeletal-muscle perfusion, sarcopenia, physical inactivity, and diminished cognitive reserve may contribute simultaneously to exercise intolerance and cognitive decline. Exercise may improve peripheral conditioning, vascular function, and neurotrophic signaling, while cognitive stimulation may promote neuroplasticity, executive functioning, and engagement with self-management. Their integration may therefore produce complementary effects on functional capacity, cognition, and adherence, although these mechanisms will not be tested as confirmatory endpoints in the present trial.

Consistent with the World Health Organization concept (22) of healthy aging, the relevance of rehabilitation in older adults extends beyond disease control to the preservation of functional ability, autonomy, emotional well-being, social participation, and quality of life. Higher physical activity levels in older adults have been associated with better self-perceived health, more frequent social relationships, and greater satisfaction with health and social care services (23). Synchronous telerehabilitation may support these goals by reducing geographical and mobility barriers while promoting functional capacity and quality of life in frail older adults (12, 24).

The TeleRiab4FRA study was designed to address this evidence gap. It evaluates an 8-week high-technology telerehabilitation program combining sensorimotor and cognitive training in frail and pre-frail older adults with chronic HF. The intervention is delivered through a synchronous digital telerehabilitation platform using CE-marked devices for remote monitoring of clinical parameters, including electrocardiographic tracing, heart rate, peripheral oxygen saturation, and blood pressure. The program is compared with a caregiver-supervised home-based exercise program matched for rehabilitation content and tailored according to the patient's frailty profile.

The primary objective of this study is to assess whether high-technology telerehabilitation improves functional capacity, measured as the change in Peak Oxygen Uptake (VO₂peak) during cardiopulmonary exercise testing (CPET), compared with caregiver-supervised home-based rehabilitation. Secondary objectives include the evaluation of frailty status, physical performance, cognitive function, health-related quality of life, treatment adherence, caregiver burden, technological usability and acceptability, and selected biomarkers associated with HF and frailty. By integrating clinical rehabilitation, digital monitoring, and home-based care, TeleRiab4FRA aims to contribute to the development of a scalable, sustainable, and patient-centered model of rehabilitation for older frail and pre-frail patients with chronic HF.

In addition to the primary hypothesis, we hypothesize that the telerehabilitation group will show greater improvement or stabilization in frailty status and physical performance, more favorable changes in cognitive function and health-related quality of life, and higher treatment adherence than the caregiver-supervised home-based group. Caregiver-related outcomes, usability, safety, and biomarkers will be interpreted as secondary or exploratory according to the prespecified analysis plan.

## 2 Methods

### 2.1 Study design and population

TeleRiab4FRA is a randomized, controlled, prospective, monocentric, parallel-group, open-label, non-profit clinical trial designed to evaluate the effectiveness of a high-technology telerehabilitation program in frail and pre-frail older adults with chronic heart failure. The study compares an 8-week synchronous sensorimotor and cognitive telerehabilitation program with a caregiver-supervised home-based rehabilitation program matched for rehabilitation content and tailored according to the patient's frailty profile.

The trial will be conducted at the Cardiac Rehabilitation Unit of the AOU “San Giovanni di Dio e Ruggi d’Aragona”, Salerno, Italy. Eligible participants will be male and female patients aged 65 years or older, with a diagnosis of Chronic Heart Failure (CHF) for at least 6 months, clinically stable and receiving optimized pharmacological therapy for at least 1 month. Patients with HF with reduced, mildly reduced, or preserved ejection fraction will be eligible, provided that they are classified as New York Heart Association (NYHA) functional class I–III and present a condition of pre-frailty or frailty.

Frailty status will be documented using validated multidimensional and functional tools, including the Fried frailty phenotype (2), the Comprehensive Geriatric Assessment (CGA) (25), the Italian Frailty Index (IFI) (26), and the Short Physical Performance Battery (SPPB) (27). Patients will be considered eligible if they meet predefined criteria for pre-frailty or frailty according to at least one of these instruments. The presence of a caregiver will be required when necessary to support safe participation in the rehabilitation program and completion of home-based activities (Table 1).

**Table 1:** Inclusion and exclusion criteria for the TeleRiab4FRA study. CGA, Comprehensive Geriatric Assessment; eGFR, estimated glomerular filtration rate; HFpEF, heart failure with preserved ejection fraction; HFmrEF, heart failure with mildly reduced ejection fraction; HFrEF, heart failure with reduced ejection fraction; IFI, Italian Frailty Index; NYHA, New York Heart Association; SPPB, Short Physical Performance Battery.

Inclusion criteria	Exclusion criteria
Age ≥ 65 years, male or female	Age < 65 years
Confirmed diagnosis of CHF for at least 6 months	NYHA class IV
Optimized and stable HF therapy for at least 1 month	Planned heart transplantation or ventricular assist device implantation within 6 months
NYHA functional class I–III	Severe renal impairment, defined as eGFR < 30 mL/min/1.73 m^2^, or dialysis dependency
Any left ventricular ejection fraction phenotype: HFrEF, HFmrEF, or HFpEF	Severe comorbidities markedly limiting life expectancy
Pre-frailty or frailty documented by at least one validated tool: Fried phenotype, IFI Index, CGA, or SPPB	Absence of pre-frailty/frailty, defined as Fried = 0, IFI = 0, and SPPB ≥ 11/12, or SPPB = 10/12 without sit-to-stand impairment
Fried phenotype: pre-frailty = 1–2 criteria; frailty = ≥ 3 criteria	SPPB < 5/12, indicating severe functional impairment not compatible with safe protocol execution
IFI ≥ 1: pre-frailty = 1–16; moderate frailty = 16.1–27; severe frailty = 27.1–40	Inability to walk independently, even with walking aids
SPPB 5–9/12 compatible with functional frailty; SPPB 10/12 compatible with pre-frailty if impairment is attributable to sit-to-stand	Severe visual impairment preventing study procedures, even with caregiver support
Ability to provide informed consent or availability of a legally authorized representative	Current or suspected pregnancy
Presence of a caregiver, when required for safe participation	Inability or unwillingness to comply with study procedures

After screening and baseline assessment, participants will be randomized in a 1:1 ratio to either the telerehabilitation group or the control group. The telerehabilitation group will receive the rehabilitation program through a synchronous digital platform with remote monitoring of clinical parameters, while the control group will perform an individualized home-based program supervised by a caregiver or family member and supported by a daily activity diary. Both groups will receive motor and cognitive training tailored according to frailty status.

Randomization will be performed after completion of the baseline assessment using a computer-generated random sequence with a 1:1 allocation ratio and permuted blocks of variable, undisclosed size. Randomization will be stratified by baseline frailty status (pre-frail versus frail) and NYHA functional class (I–II versus III). The allocation sequence will be generated by an independent biostatistician or Statistical Unit using validated software and will not be accessible to personnel involved in screening or enrolment. Allocation concealment will be ensured using sequentially numbered, opaque, sealed envelopes prepared by the Statistical Unit, with assignment traceability maintained through an audit trail. Any deviation from the allocation procedure will be documented in the CRF. Owing to the nature of the intervention, participants and treating professionals cannot be blinded; whenever feasible, endpoint assessment and statistical analysis will be performed by personnel not involved in treatment delivery using coded datasets.

**Figure 1:**
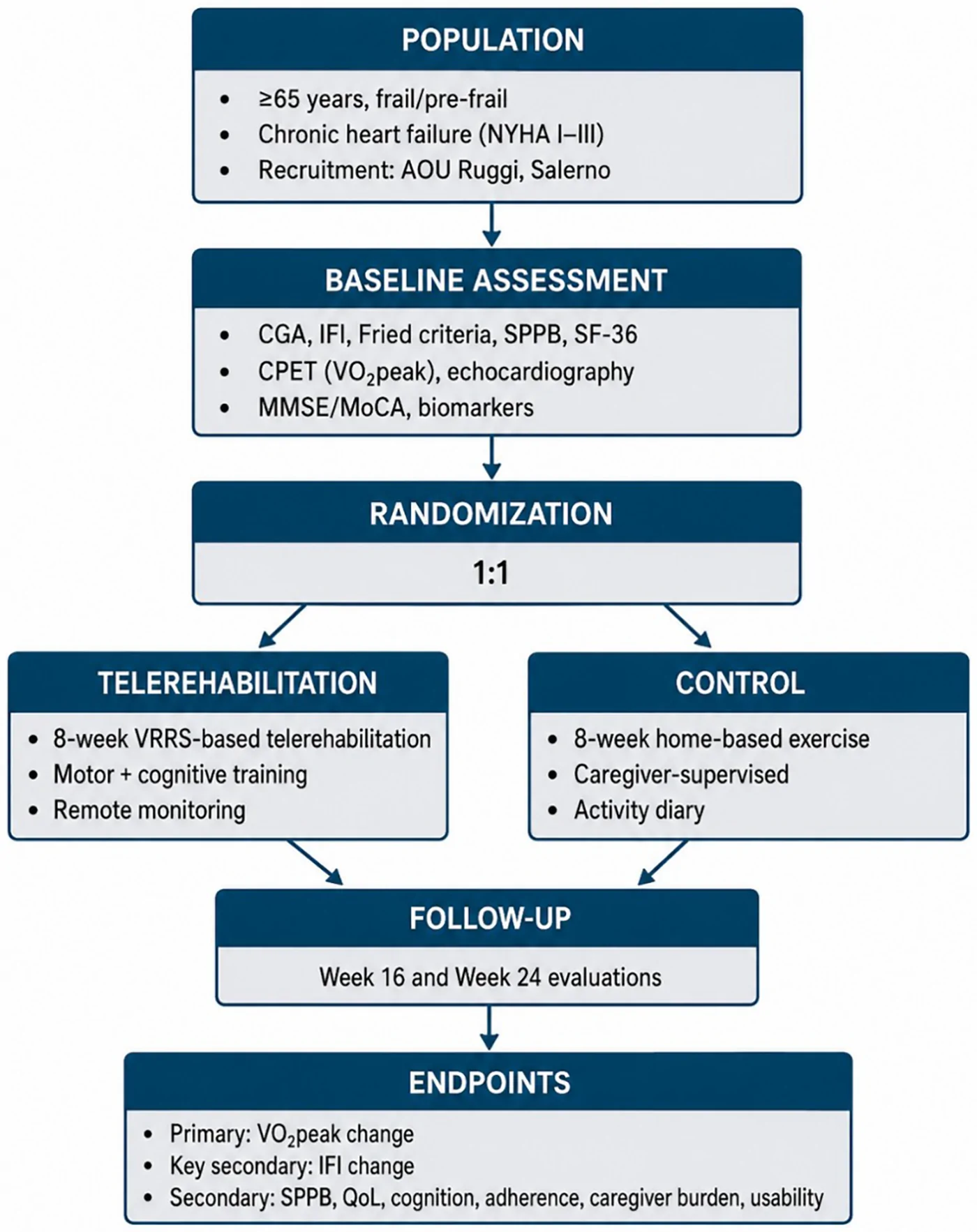
Study flow diagram of the TeleRiab4FRA randomized controlled trial. The figure summarizes the study population, baseline assessment, 1:1 randomization, intervention and control arms, follow-up schedule, and main study endpoints. CGA, Comprehensive Geriatric Assessment; CPET, cardiopulmonary exercise testing; IFI, Italian Frailty Index; MMSE, Mini-Mental State Examination; MoCA, Montreal Cognitive Assessment; NYHA, New York Heart Association; QoL, quality of life; SPPB, Short Physical Performance Battery; VO₂peak, peak oxygen uptake; VRRS, Virtual Reality Rehabilitation System.

Study assessments will be performed at baseline (T0), at 4 weeks (T1), at the end of the 8-week intervention (T2), at 16 weeks (T3), and at 24 weeks (T4). The primary post-treatment assessment will be conducted at T2. After completion of the assigned 8-week rehabilitation program, no additional study-prescribed rehabilitation sessions will be delivered between weeks 9 and 24; participants in both groups will continue optimized medical therapy and usual clinical care, and any non-study rehabilitation or structured physical activity initiated during follow-up will be recorded. At T3 (week 16), participants will undergo an intermediate follow-up assessment including IFI, SPPB, MMSE, MoCA, adherence to any self-directed physical activity, adverse events, and relevant clinical changes. At T4 (week 24), the main follow-up assessment will include CPET-derived VO₂peak, IFI, SPPB, SF-36, MMSE, MoCA, BNP, ANP, adverse events, and relevant clinical changes to evaluate maintenance of treatment effects. The study population is expected to include approximately 80 participants overall, allowing for the required sample size for the primary endpoint and an anticipated drop-out rate (Figure 1).

### 2.2 Groups and interventions

After completion of the baseline assessment, participants will be randomly assigned to one of two study arms: the telerehabilitation group or the control group. Both groups will receive an 8-week individualized rehabilitation program including motor and cognitive training. The rehabilitation content will be tailored according to each participant's frailty profile, while the main difference between groups will be the mode of delivery, supervision, and monitoring.

#### 2.2.1 Telerehabilitation group

Participants allocated to the telerehabilitation group will receive an 8-week synchronous high-technology telerehabilitation program delivered through a CE-marked digital platform. The intervention will combine sensorimotor and cognitive training and will be remotely supervised by qualified healthcare professionals. Before starting the home-based telerehabilitation program, patients and caregivers will receive in-person training on the use of the platform and devices, together with technical instructions and safety procedures.

The telerehabilitation system will allow real-time audio-video interaction between the patient and the rehabilitation team, remote supervision of exercise execution, and monitoring of relevant clinical parameters, including electrocardiographic tracing, heart rate, peripheral oxygen saturation, and blood pressure. Remote monitoring will be used to support safety during the sessions, guide exercise progression, and identify any clinical or technical issue requiring intervention. In case of abnormal clinical parameters, symptoms, or difficulties in performing the exercises, the session may be interrupted and the patient may be referred for additional clinical evaluation.

The motor training program will last approximately 60 min per session, including warm-up, active training, and cool-down. Exercise intensity will be individualized according to baseline CPET findings, with a target progressing toward approximately 70% of baseline VO₂peak; when CPET-based prescription is not feasible, 75%–80% of the maximum heart rate recorded during ergometric testing will be used. The prescription will be progressively adapted to exercise tolerance, clinical status, and frailty level. The cognitive training component will last approximately 30 min per session and will include exercises targeting memory, visuospatial abilities, executive functions, and psychomotor speed.

Training load, task complexity, and exercise duration may be increased when the participant completes the prescribed activities with correct execution, acceptable perceived exertion, stable symptoms, and physiological values within the study safety thresholds. The load will be maintained, reduced, or the session interrupted in the presence of excessive Borg-rated exertion, relevant symptoms, abnormal vital signs, unsafe movement execution, or incomplete recovery. All adaptations and their reasons will be recorded by the treating professional.

Treatment frequency will be adapted according to frailty level. Pre-frail patients, defined by an IFI score between 1 and 16, will perform 2–4 sessions per week during the first 4 weeks and 3–5 sessions per week from week 5 to week 8. Frail patients, defined by an IFI score between 16.1 and 40, will perform 3–5 sessions per week throughout the 8-week program.

#### 2.2.2 Control group

Participants allocated to the control group will receive an individualized home-based rehabilitation program supervised by a caregiver or family member. The program will be initially explained in person by the rehabilitation team and will include motor and cognitive exercises matched to the patient's frailty profile and comparable in content and duration to those prescribed for the telerehabilitation group.

The motor component will last approximately 60 min per session, including warm-up, active exercise, and cool-down. The cognitive component will last approximately 30 min per session and will include exercises targeting the same cognitive domains addressed in the telerehabilitation group. Treatment frequency will follow the same frailty-based schedule used for the telerehabilitation group: 2–4 sessions per week during the first 4 weeks and 3–5 sessions per week from week 5 to week 8 for pre-frail patients, and 3–5 sessions per week throughout the 8-week program for frail patients.

Caregivers will receive standardized in-person training on the prescribed exercises, session schedule, safe assistance, recognition of warning symptoms, measurement of basic clinical parameters when required, and procedures for contacting the clinical team. Before home treatment begins, caregiver understanding and practical competence will be verified using a structured checklist. Participants and caregivers will receive a written exercise schedule and illustrated instructions and will complete a daily activity diary documenting exercises performed, session duration, perceived exertion and tolerance, symptoms, adverse events, and any deviations from the prescribed program. A member of the rehabilitation team will conduct a structured weekly telephone or video contact to review the diary, verify completion and quality of the sessions, reinforce correct exercise execution, resolve doubts, and document protocol deviations. Diaries will be reviewed at weeks 4 and 8, and fidelity will be summarized as the proportion of prescribed sessions completed and the proportion performed according to the planned duration and exercise content.

#### 2.2.3 Intervention standardization and safety

In both groups, the rehabilitation program will be individualized according to baseline clinical status, frailty level, functional capacity, and exercise tolerance. A common intervention manual will define the motor and cognitive content, target session duration, frailty-based frequency, progression criteria, warning signs, and permitted adaptations. This approach is intended to maintain comparability of rehabilitation dose and content while preserving the specific features of each delivery model. Fidelity in the telerehabilitation group will be assessed through platform logs, session attendance, sensor-derived performance data, and therapist records; fidelity in the control group will be assessed through caregiver competency checks, weekly structured contacts, and review of daily diaries. Participants will continue optimized medical therapy and usual care for CHF throughout the study period.

Safety procedures will be applied in both groups, and both patients and caregivers will be instructed to stop exercise in case of chest pain, marked dyspnea, dizziness, excessive fatigue, palpitations, abnormal vital parameters, or any other concerning symptom. Any adverse event, protocol deviation, or clinically relevant issue occurring during the intervention period will be recorded in the study Case Report Form (CRF) and managed according to Good Clinical Practice principles and local clinical procedures (Table 2).

**Table 2:** Comparison between the telerehabilitation and control interventions.

Feature	Telerehabilitation group	Control group
Intervention duration	8 weeks	8 weeks
Setting	Home-based, synchronous telerehabilitation	Home-based rehabilitation
Delivery mode	Real-time supervision through a digital telerehabilitation platform	Exercises initially explained in person and subsequently performed at home
Supervision	Remote supervision by qualified healthcare professionals	Supervision by caregiver or family member
Rehabilitation components	Motor training and cognitive training	Motor training and cognitive training
Motor training duration	Approximately 60 min per session, including warm-up, active training, and cool-down	Approximately 60 min per session, including warm-up, active training, and cool-down
Cognitive training duration	Approximately 30 min per session	Approximately 30 min per session
Exercise personalization	Tailored according to clinical status, frailty level, baseline functional capacity, and exercise tolerance	Tailored according to clinical status, frailty level, baseline functional capacity, and exercise tolerance
Frequency in pre-frail patients	2–4 sessions/week during weeks 1–4; 3–5 sessions/week during weeks 5–8	2–4 sessions/week during weeks 1–4; 3–5 sessions/week during weeks 5–8
Frequency in frail patients	3–5 sessions/week throughout the 8-week program	3–5 sessions/week throughout the 8-week program
Monitoring	Remote monitoring of ECG, heart rate, peripheral oxygen saturation, and blood pressure	Measurement of basic clinical parameters when required
Technology	CE-marked digital telerehabilitation platform with connected devices and remote monitoring tools	No digital telerehabilitation platform; use of diary and caregiver support
Adherence assessment	Platform-based monitoring and session attendance	Daily activity diary and reported session completion
Safety procedures	Real-time supervision, remote monitoring, session interruption in case of warning signs or abnormal parameters	Caregiver instruction, symptom monitoring, interruption of exercise in case of warning signs
Caregiver role	Support in device use and home setting, when needed	Direct supervision of home-based sessions
Main difference between groups	Digital synchronous delivery with remote professional supervision and monitoring	Conventional home-based delivery supervised by caregiver/family member

### 2.3 Technological solution

The telerehabilitation intervention will be delivered using the Khymeia Virtual Reality Rehabilitation System (VRRS; Khymeia Group, Noventa Padovana, Italy), a CE-marked Class I medical device designed to support technology-assisted rehabilitation in clinical and home settings (28–34). In TeleRiab4FRA, VRRS will be configured as a hub-and-spoke architecture in which the clinical TeleCockpit represents the professional hub and each VRRS Home Kit represents a patient-side node. The system will support synchronous, remotely supervised sensorimotor and cognitive rehabilitation, physiological monitoring, treatment assignment, adherence tracking, and review of performance data (Table 3).

**Table 3:** Components and functions of the telerehabilitation system used in the TeleRiab4FRA study. BP, blood pressure; ECG, electrocardiographic; HR, heart rate; K-RING, Khymeia Ring sensor; K-SENSOR, Khymeia inertial sensor; K-SPIRO, Khymeia spirometer; SpO₂, peripheral oxygen saturation; VRRS, Virtual Reality Rehabilitation System.

Component	Main function	Use in the TeleRiab4FRA study
VRRS platform	Digital rehabilitation environment for remote delivery of personalized exercises	Delivery of synchronous sensorimotor and cognitive telerehabilitation sessions
TeleCockpit station	Professional workstation for remote session management and clinical supervision	Used by the rehabilitation team to supervise patients, manage sessions, and monitor rehabilitation activities
VRRS Home Kit	Home-based telerehabilitation kit	Enables patients to perform supervised rehabilitation sessions at home
Tablet device	Patient-side interface for audio-video communication and exercise delivery	Allows real-time interaction between patient and rehabilitation professional
K-SENSOR	Inertial sensors for movement tracking	Monitors movement execution, exercise performance, and adherence during motor training
K-Sensor bands	Supports correct positioning of inertial sensors	Facilitates accurate and reproducible sensor placement on the patient's limbs
K-RING	Wearable sensor for heart rate and peripheral oxygen saturation monitoring	Supports physiological monitoring during home-based sessions
K-SPIRO	Spirometer for guided respiratory exercises	Used for breathing exercises and respiratory components of the rehabilitation program
Health Monitor	Device for measuring and storing blood pressure and single-lead electrocardiographic data	Supports cardiovascular monitoring and safety assessment during the telerehabilitation pathway
Kloud service module	Web-based service for data sharing, treatment assignment, and remote monitoring	Allows centralized management of rehabilitation protocols, session data, adherence, and patient progress
Remote technical support	Assistance for device use and troubleshooting	Supports patients and caregivers in case of technical or connectivity issues

Before starting the home-based program, participants allocated to the telerehabilitation group will complete an initial in-person familiarization phase. During this phase, patients and caregivers will be trained in the use of the platform, wearable devices, respiratory tools, and safety procedures. This preliminary training will be used to verify the patient's ability to interact with the system, ensure correct device placement, and identify the need for caregiver support during the home-based intervention.

The technological infrastructure includes a professional TeleCockpit station used by the rehabilitation team to schedule and manage sessions, assign individualized exercises, establish encrypted audio-video communication, remotely supervise patients, and visualize incoming physiological and performance data. The patient-side configuration includes a VRRS Home Kit with a tablet-based interface and connected sensors. During a synchronous session, the prescribed exercise is transmitted to the patient interface; movement and physiological signals are acquired by the connected devices and made available to the supervising professional through the platform. Session metadata, completion status, exercise parameters, and available sensor outputs are stored within the system and can subsequently be reviewed through the Kloud service module. The architecture therefore supports a bidirectional data flow: treatment instructions and parameter updates move from the clinician to the home unit, while adherence, performance, symptoms, and physiological measurements move from the home unit to the clinical workstation.

The VRRS Home Kit includes inertial sensors for movement tracking, wearable sensors for heart rate and peripheral oxygen saturation, a spirometer for guided respiratory exercises, and a health monitor for blood pressure and single-lead electrocardiographic data. Movement sensors provide information on exercise execution and task completion, whereas physiological devices support safety surveillance and evaluation of exercise response. Data are reviewed in real time during synchronous sessions when technically available; session records and stored measurements can also be reviewed after the session. The platform does not autonomously diagnose clinical deterioration: all alerts, abnormal values, or performance concerns are interpreted by qualified healthcare professionals according to the study safety procedures.

Personalization is clinician-driven and follows prespecified clinical rules. At baseline, the rehabilitation team selects the motor and cognitive modules, session frequency, task complexity, exercise intensity, and progression targets according to CPET results, NYHA class, frailty profile, SPPB performance, cognitive status, comorbidities, and exercise tolerance. During the intervention, the clinician may progress, maintain, simplify, or interrupt an activity on the basis of observed execution, Borg-rated exertion, symptoms, vital-sign response, completion rate, and changes in functional status. Modifications are documented in the treatment record. Thus, the platform functions as a delivery, monitoring, and decision-support environment, while responsibility for personalization remains with the rehabilitation professional.

To improve usability in older frail and pre-frail patients, the technological pathway includes structured patient and caregiver education, simplified instructions for device use, and remote technical support when required. In case of technical issues, poor connectivity, difficulty in using the devices, abnormal clinical parameters, or symptoms suggestive of clinical instability, the session can be interrupted and the patient can be contacted for further assessment. If needed, the patient may be referred for in-person clinical evaluation to ensure safety and continuity of care.

All data collected through the telerehabilitation platform will be managed according to applicable data protection regulations and stored in a secure digital environment. Access will be role-based and restricted to authorized members of the clinical and research teams. Patient identifiers used for clinical management will be separated from the coded research dataset used for analysis. The platform records session dates, assigned activities, completion, available sensor measurements, and technical or clinical interruptions, thereby supporting traceability, adherence assessment, and audit of intervention fidelity.

### 2.4 Outcomes

The outcome measures of the TeleRiab4FRA study were defined to provide a multidimensional evaluation of the effects of high-technology telerehabilitation in frail and pre-frail older adults with CHF. Outcomes include functional capacity, frailty status, physical performance, health-related quality of life, cognitive function, biochemical parameters, caregiver-related outcomes, technological usability, treatment adherence, and safety. All outcomes will be collected according to the pre-specified assessment schedule and in line with the registered trial protocol.

The primary outcome is the change in functional capacity from baseline to the end of the 8-week intervention, measured as VO₂peak during CPET (35, 36). The primary efficacy analysis will compare VO₂peak at 8 weeks between the telerehabilitation group and the control group, adjusted for baseline values. VO₂peak will also be assessed at the 24-week follow-up to evaluate the maintenance of treatment effects over time (37).

The key secondary outcome is the change in frailty status assessed using the IFI (26), which will be collected at baseline, 4 weeks, 8 weeks, 16 weeks, and 24 weeks, and changes over time will be compared between the telerehabilitation and control groups.

Physical performance will be assessed using the SPPB, which includes balance, gait speed, and chair stand tests (38, 39). The SPPB provides a composite score ranging from 0 to 12, with higher scores indicating better lower-extremity function and physical performance. SPPB will be assessed at baseline, 4 weeks, 8 weeks, 16 weeks, and 24 weeks.

Health-related quality of life will be evaluated using the Short Form-36 Health Survey (SF-36), which assesses multiple domains, including physical functioning, role limitations, bodily pain, general health, vitality, social functioning, emotional well-being, and mental health (40, 41). Scores range from 0 to 100, with higher scores indicating better perceived health status and quality of life. SF-36 will be administered at baseline, 8 weeks, and 24 weeks.

Cognitive function will be evaluated using both the Mini-Mental State Examination (MMSE) (42) and the Montreal Cognitive Assessment (MoCA) (43). The MMSE assesses global cognitive function, including orientation, attention, memory, language, and visuospatial abilities, with total scores ranging from 0 to 30 and higher scores indicating better cognitive performance (44). The MoCA will be used to provide an additional assessment of cognitive domains relevant to executive function, attention, memory, and self-management (45). Both MMSE and MoCA will be collected at baseline, 4 weeks, 8 weeks, 16 weeks, and 24 weeks.

Biochemical parameters will include B-type natriuretic peptide (BNP) and atrial natriuretic peptide (ANP), expressed in picograms per milliliter (46). Changes in BNP and ANP levels will be assessed at baseline, 8 weeks, and 24 weeks to explore potential biological changes associated with the rehabilitation program and HF status.

Caregiver-related outcomes will include caregiver burden and perceived stress. Caregiver burden will be assessed using the 12-item Zarit Burden Interview (ZBI-12) (47), while perceived stress will be assessed using the 10-item Perceived Stress Scale (PSS-10) (48). Both instruments will be administered at baseline and at 8 weeks to explore the impact of the rehabilitation pathway on caregivers.

Technological usability and acceptability will be evaluated only in the telerehabilitation group using the System Usability Scale (SUS) (49), a 10-item questionnaire with total scores ranging from 0 to 100, where higher scores indicate better perceived usability. SUS will be administered at the end of the 8-week intervention.

Treatment adherence will be assessed as the percentage of scheduled motor and cognitive training sessions completed during the 8-week intervention period. In the telerehabilitation group, adherence and fidelity will be documented through platform-based session records, attendance, therapist records, and available sensor-derived performance data. In the control group, adherence and fidelity will be assessed through daily activity diaries, caregiver competency verification, structured weekly contacts, and review of whether the prescribed frequency, duration, and exercise content were followed. Major deviations and reasons for missed or modified sessions will be recorded in the CRF.

Safety outcomes will include adverse events, symptoms occurring during exercise, abnormal clinical parameters, technical issues affecting session delivery, and any interruption or modification of the rehabilitation program. Safety information will be collected throughout the intervention and follow-up period (Table 4).

**Table 4:** TeleRiab4FRA outcome measures by assessment timepoint. ANP, atrial natriuretic peptide; BNP, B-type natriuretic peptide; IFI, Italian Frailty Index; MMSE, Mini-Mental State Examination; MoCA, Montreal Cognitive Assessment; PSS-10, 10-item Perceived Stress Scale; SF-36, Short Form-36 Health Survey; SPPB, Short Physical Performance Battery; SUS, System Usability Scale; VO₂peak, peak oxygen uptake; ZBI-12, 12-item Zarit Burden Interview.

Outcome type	Outcome measure	Domain	Baseline	4 weeks	8 weeks	16 weeks	24 weeks
Primary	Change in VO₂peak	Functional capacity	✓		✓		✓
Secondary, key	Change in IFI	Frailty status	✓	✓	✓	✓	✓
Secondary	Change SPPB score	Physical performance	✓	✓	✓	✓	✓
Secondary	Change in SF-36 score	Quality of life	✓		✓		✓
Secondary	Change in MMSE score	Cognitive function	✓	✓	✓	✓	✓
Secondary	Change in MoCA score	Cognitive function	✓	✓	✓	✓	✓
Secondary	Change in BNP levels	Biochemical	✓		✓		✓
Secondary	Change in ANP levels	Biochemical	✓		✓		✓
Secondary	Change in ZBI-12 score	Caregiver burden	✓		✓		
Secondary	Change in PSS-10 score	Caregiver stress	✓		✓		
Secondary	SUS score	Usability and acceptability			✓		
Secondary	Treatment adherence	Process measure		✓	✓		
Safety	Adverse events	Safety and feasibility		✓	✓	✓	✓

SUS will be administered only to participants allocated to the telerehabilitation group. Treatment adherence will be assessed throughout the 8-week intervention period as the percentage of scheduled motor and cognitive training sessions completed. The 24-week assessment of VO₂peak will be used to evaluate maintenance of treatment effects over time and will not replace the primary post-treatment analysis at 8 weeks.

### 2.5 Safety management and contingency planning

A structured safety management and contingency plan will be implemented to minimize clinical and operational risks during the TeleRiab4FRA study. The safety procedures will apply to both study groups, with specific measures adapted to the mode of delivery of each intervention.

In the telerehabilitation group, all sessions will be delivered synchronously and remotely supervised by qualified healthcare professionals. During each session, the rehabilitation team will monitor the patient's clinical status, exercise execution, and physiological response to training. The telerehabilitation platform will support remote monitoring of relevant clinical parameters, including heart rate, peripheral oxygen saturation, blood pressure, and single-lead electrocardiographic data. These data will be used to guide exercise progression, verify tolerance, and identify potential warning signs.

Before starting the home-based program, patients and caregivers will receive in-person training on the use of the telerehabilitation devices, correct sensor placement, recognition of warning symptoms, and procedures to follow in case of clinical or technical problems. Patients will be instructed to stop exercise and immediately inform the rehabilitation team in case of chest pain, marked dyspnea, dizziness, palpitations, excessive fatigue, neurological symptoms, altered consciousness, falls, or any other concerning symptom.

The rehabilitation session will be interrupted in the presence of abnormal clinical parameters, relevant symptoms, unsafe exercise execution, or technical issues that prevent adequate supervision. In such cases, the patient will be contacted by the rehabilitation team and, when necessary, referred for cardiological evaluation, in-person rehabilitation assessment, or emergency medical care according to local clinical procedures.

In the control group, patients will perform the home-based program under the supervision of a caregiver or family member. Caregivers will receive specific instructions on how to support the patient during the sessions, recognize signs of intolerance, measure basic clinical parameters when required, interrupt exercise in case of warning symptoms, and contact the clinical team if clinical concerns arise. The daily activity diary will also be used to record session completion, perceived tolerance, symptoms, and any adverse events.

Technical contingency procedures will be applied in the telerehabilitation group. In case of device malfunction, connectivity problems, difficulties in using the platform, or inadequate data transmission, remote technical assistance will be provided. If the problem cannot be resolved and safe remote supervision cannot be ensured, the session will be postponed, modified, or replaced by an in-person assessment or session, according to clinical judgement and organizational feasibility.

**Figure 2:**
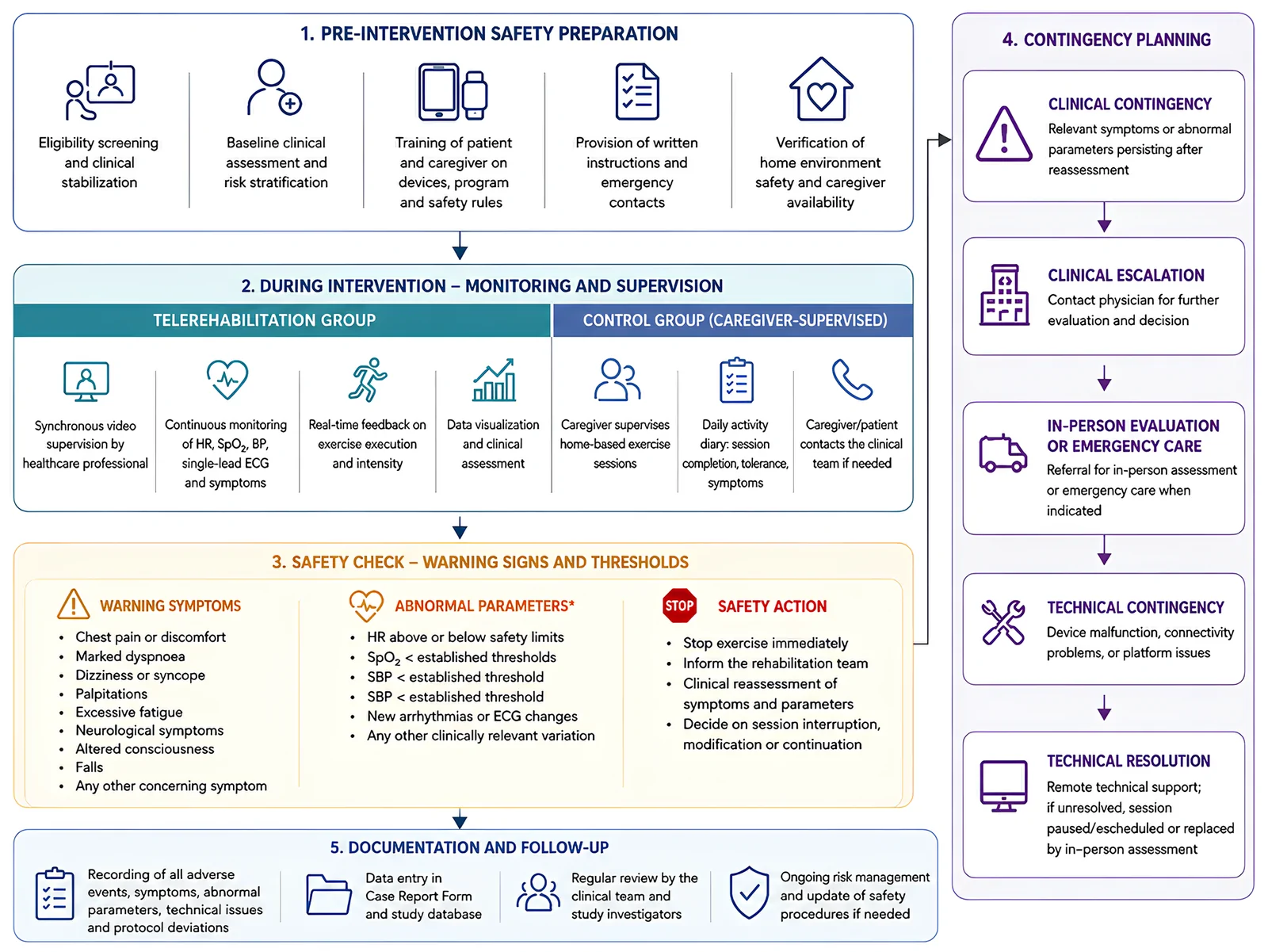
Safety thresholds and actions. BP, blood pressure; ECG, electrocardiogram; HR, heart rate; SBP, systolic blood pressure; SpO₂, peripheral oxygen saturation.

All adverse events, symptoms occurring during exercise, abnormal parameters, technical issues, protocol deviations, and interruptions of the rehabilitation program will be documented in the CRF. Serious adverse events will be managed and reported according to Good Clinical Practice principles, local institutional procedures, and applicable regulatory requirements. This safety framework is intended to ensure patient protection, therapeutic continuity, and traceability of clinical and technical events throughout the study (Figure 2).

### 2.6 Statistical analysis

All statistical analyses will be performed according to a pre-specified Statistical Analysis Plan before database lock. Descriptive statistics will be used to summarize baseline demographic, clinical, functional, cognitive, biochemical, and caregiver-related characteristics. Continuous variables will be reported as mean and standard deviation or median and interquartile range, according to data distribution. Categorical variables will be summarized as absolute frequencies and percentages. Normality will be assessed using graphical methods and appropriate statistical tests.

The primary analysis will be conducted according to the intention-to-treat (ITT) principle, including all randomized participants and analyzing them according to their original allocation group. A per-protocol (PP) analysis will also be performed as a sensitivity analysis, including participants who complete at least 80% of the scheduled motor and cognitive training sessions without major protocol deviations. The safety population will include all participants who start the assigned rehabilitation program and will be used for the description of adverse events and safety-related outcomes.

#### 2.6.1 Primary endpoint analysis

The primary endpoint is the change in functional capacity from baseline to the end of the 8-week intervention, assessed by VO₂peak measured during cardiopulmonary exercise testing.

The primary efficacy analysis will compare VO₂peak at 8 weeks between the telerehabilitation group and the control group using analysis of covariance (ANCOVA), with treatment group as the fixed factor and baseline VO₂peak as the mandatory covariate. Additional pre-specified covariates may include age, sex, NYHA class, and baseline frailty level, if clinically relevant or imbalanced between groups at baseline. The treatment effect will be expressed as adjusted mean difference between groups, with 95% confidence interval and *p*-value.

As a supportive analysis, the absolute change in VO₂peak from baseline to 8 weeks will also be calculated and compared between groups. A longitudinal analysis will be performed using a linear mixed model for repeated measures, including time, group, and group-by-time interaction as fixed effects and participant as a random effect. This model will allow the evaluation of VO₂peak trajectories over time, including baseline, post-treatment, and follow-up assessments. The 24-week assessment will be used to evaluate the maintenance of treatment effects and will be interpreted as a follow-up analysis rather than as the primary confirmatory endpoint.

#### 2.6.2 Secondary endpoint analysis

Secondary outcomes will be analyzed according to their measurement scale and distribution. Continuous outcomes, including IFI, SPPB, SF-36, MMSE, MoCA, BNP, ANP, ZBI-12, and PSS-10 scores, will be summarized at each timepoint and analyzed using ANCOVA models adjusted for the corresponding baseline value when post-treatment comparisons between groups are performed.

When repeated measurements are available across multiple timepoints, linear mixed models will be used to assess changes over time and group-by-time interactions. These models will include all available observations and will allow the evaluation of outcome trajectories across baseline, 4 weeks, 8 weeks, 16 weeks, and 24 weeks, when applicable.

The Italian Frailty Index will be considered the key secondary endpoint. Other clinically relevant secondary outcomes will include SPPB, SF-36, and treatment adherence. For this pre-defined set of clinically relevant secondary outcomes, adjustment for multiple comparisons will be performed using the Holm-Bonferroni procedure, with an overall significance level of 0.05. All other secondary and exploratory outcomes, including cognitive measures, biochemical parameters, caregiver-related measures, and technological usability, will be interpreted primarily in a descriptive and exploratory manner, with estimates of effect, 95% confidence intervals, and *p*-values reported where appropriate.

Treatment adherence will be calculated as the percentage of scheduled motor and cognitive sessions completed during the 8-week intervention period. Between-group differences in adherence will be analyzed descriptively and, where appropriate, using parametric or non-parametric tests according to data distribution. Usability, assessed using the System Usability Scale in the telerehabilitation group only, will be reported descriptively as mean, standard deviation, median, interquartile range, and score distribution.

Safety outcomes, including adverse events, symptoms during exercise, abnormal clinical parameters, technical issues, session interruptions, and protocol deviations, will be summarized by group using frequencies, percentages, and event rates where appropriate.

Predefined exploratory subgroup analyses will examine whether treatment effects vary according to baseline frailty status (pre-frail versus frail, and where appropriate moderate versus severe frailty), NYHA functional class (I–II versus III), sex, and age (for example, ≤80 versus >80 years). Treatment-by-subgroup interaction terms may be estimated within the relevant ANCOVA or mixed-effects models. Because the trial is not powered for subgroup effects, these analyses will be considered hypothesis-generating and interpreted cautiously using interaction estimates and 95% confidence intervals.

#### 2.6.3 Sample size calculation

The sample size was calculated on the basis of the primary endpoint, defined as the change in functional capacity measured by VO₂peak during cardiopulmonary exercise testing from baseline to the end of the 8-week intervention.

A between-group difference of 2.5 mL/kg/min in VO₂peak (50) was considered clinically meaningful. Assuming a standard deviation of 3.5 mL/kg/min, corresponding to a Cohen's d effect size of approximately 0.71, the sample size calculation was performed using G*Power software, based on a two-tailed test for the difference between two independent means, with a significance level of *α* = 0.05, statistical power of 80%, and 1:1 allocation.

The calculation indicated that 33 participants per group, corresponding to 66 participants overall, are required to detect the expected difference in the primary endpoint. Considering an anticipated dropout rate of approximately 15%–20%, the study plans to enroll approximately 80 participants overall, in order to preserve adequate statistical power for the primary efficacy analysis.

#### 2.6.4 Handling of missing data and dropouts

Missing data will be described by treatment group, outcome, and assessment timepoint. The amount, pattern, and potential reasons for missingness will be examined to evaluate whether missing data are likely to be missing completely at random, missing at random, or not missing at random.

The primary analysis will be conducted on the ITT population. Linear mixed models for repeated measures will be used as part of the analytical strategy because they allow inclusion of participants with partially observed follow-up data under the missing at random assumption. If the proportion of missing data at the primary post-treatment assessment is relevant or unbalanced between groups, sensitivity analyses using multiple imputation will be performed. The imputation model may include treatment group, baseline value of the outcome, age, sex, NYHA class, frailty level, and other variables associated with missingness or outcome values.

Dropouts and protocol deviations will be documented in the CRF, including the timing and reason for withdrawal whenever available. Participants who discontinue the intervention but remain available for outcome assessment will be invited to complete follow-up evaluations according to the original study schedule. Per-protocol analyses will be conducted as sensitivity analyses to assess the robustness of the primary findings among participants with adequate intervention exposure and without major protocol deviations.

## 3 Expected results

We hypothesize that participants allocated to the telerehabilitation group will show greater improvement in functional capacity, measured by VO₂peak during cardiopulmonary exercise testing, at the end of the 8-week intervention compared with participants receiving caregiver-supervised home-based rehabilitation.

We also expect the telerehabilitation group to show favorable changes or stabilization in frailty status and physical performance, as assessed by the IFI and the SPPB. These changes would suggest a potential positive effect of an integrated sensorimotor and cognitive rehabilitation program on the multidimensional vulnerability of frail and pre-frail patients with CHF.

Potential improvements in health-related quality of life and cognitive function are also expected. In particular, changes in SF-36, MMSE, and MoCA scores may indicate that a structured rehabilitation program combining physical exercise, cognitive stimulation, and remote professional supervision can influence outcomes beyond cardiorespiratory fitness alone.

We expect that synchronous supervision, remote monitoring, and real-time feedback may support treatment adherence and safe execution of home-based rehabilitation. The telerehabilitation platform may also facilitate the early identification of symptoms, abnormal clinical parameters, technical problems, or difficulties in exercise execution.

Caregiver-related outcomes, technological usability, and biochemical parameters will be interpreted as exploratory. Changes in ZBI-12 and PSS-10 scores may provide preliminary information on the indirect impact of the rehabilitation pathway on caregivers. SUS scores will help assess the acceptability of the technology in an older frail population. Changes in BNP and ANP levels may generate hypotheses on the biological response to rehabilitation in patients with CHF and frailty.

Overall, the study is expected to provide preliminary evidence on whether high-technology telerehabilitation can represent a feasible, safe, and clinically meaningful home-based rehabilitation model for frail and pre-frail older adults with CHF.

## 4 Discussion

The TeleRiab4FRA study addresses an important gap in digital rehabilitation research by focusing on frail and pre-frail older adults with CHF, a population that is clinically relevant but often underrepresented in rehabilitation trials (7, 20). Previous studies have demonstrated the feasibility of remotely delivered rehabilitation in patients with heart failure and in older or frail populations, but these interventions have generally focused on exercise training, physiological telemonitoring, telephone or video coaching, remote patient management, or functional recovery in specific post-acute conditions.

For example, Bernocchi et al. evaluated an integrated home-based program combining exercise, cardiorespiratory monitoring, nursing telephone support, and physiotherapist supervision in older patients with coexisting COPD and CHF (15). Lundgren et al. investigated real-time, high-intensity home-based exercise in patients with chronic HF unable or unwilling to participate in outpatient cardiac rehabilitation (16) More recently, the Tele-ADHF trial examined multidisciplinary cardiac telerehabilitation after hospitalization for acute decompensated heart failure, comparing telerehabilitation plus remote patient management with remote patient management alone (17). These studies support the feasibility and potential clinical value of telerehabilitation but differ from TeleRiab4FRA in terms of population, intervention structure, comparator, and outcome framework.

The novelty of TeleRiab4FRA therefore does not rely on a single technological or rehabilitative component, but on the specific integration of several elements within a randomized cardiac rehabilitation protocol. First, the study targets older adults with clinically stable CHF and documented frailty or pre-frailty, rather than a general HF population or a specifically post-acute cohort. Second, frailty is used not only as an eligibility or outcome criterion, but also as a determinant of treatment frequency, progression, and personalization. This is relevant because previous programmes have often considered frailty primarily as a risk condition, a barrier to access, or a descriptive characteristic, rather than as a variable directly informing rehabilitation dose.

The main scientific contribution of this protocol is the integration of frailty assessment into a multidimensional cardiac telerehabilitation pathway. Rather than evaluating HF rehabilitation only through conventional cardiorespiratory outcomes, TeleRiab4FRA combines VO₂peak with multidimensional measures such as the IFI, SPPB, cognitive screening tools, quality of life, caregiver-related scales, adherence, usability, and safety. This approach may help clarify whether improvements in aerobic capacity are accompanied by changes in broader domains that are particularly relevant in older frail patients.

A further relevant contribution is the comparison between two structured home-based rehabilitation models. The control group receives caregiver-supervised home-based rehabilitation, while the intervention group receives comparable intended rehabilitation content and treatment frequency through a synchronous digital platform with remote professional supervision and clinical monitoring.

This active-comparator design differs from studies in which telerehabilitation has been compared with usual care, no structured rehabilitation, or remote patient management alone. For example, Lundgren et al. compared synchronous home-based exercise with standard care (51), whereas Tele-ADHF compared telerehabilitation plus remote patient management with remote patient management alone (17). The TeleRiab4FRA design is therefore intended to isolate more specifically the added value of synchronous professional supervision, sensor-based physiological and movement monitoring, immediate feedback, and platform-supported personalization beyond the effects of home exercise itself.

The intervention also reflects a multidimensional rehabilitation model. The combination of motor, cognitive, and respiratory training is particularly relevant in frail patients with CHF, in whom physical vulnerability, cognitive difficulties, reduced self-management capacity, and dependence on caregivers may coexist.

Previous work in frail populations has shown increasing interest in multicomponent exercise, comprehensive geriatric assessment, and functional personalization. The ActiveFLS protocol, for example, combines individualized exercise with comprehensive geriatric assessment, nutritional intervention, polypharmacy review, and management of cognitive and mood-related conditions (52). Its technological delivery, however, is based mainly on an asynchronous smartphone application and addresses older adults after hip fracture rather than patients with CHF.

Similarly, the Vivifrail programme adapts resistance, balance, flexibility, and endurance exercises to the participant's functional capacity (52). These approaches demonstrate that multicomponent and functionally tailored exercise is not in itself new, but they have generally been developed outside the specific context of cardiac telerehabilitation in frail and pre-frail patients with CHF.

Synchronous telerehabilitation has also been proposed as an alternative to face-to-face physiotherapy in frail older adults. Cigarroa et al., for example, compare remotely supervised videoconference sessions with an in-person multicomponent exercise programme, using primarily strength, balance, fitness, functional status, and quality-of-life outcomes (24).

TeleRiab4FRA extends this perspective by integrating structured cognitive training and repeated cognitive assessment within a cardiac telerehabilitation programme. This is particularly relevant because cognitive frailty is multidimensional and may involve physical, cognitive, psychosocial, and self-management components. A scoping review by Md Fadzil et al. showed that telerehabilitation studies in older adults with mild cognitive impairment or cognitive frailty remain limited and frequently rely on smartphones, web-based tools, videoconferencing, or television-based technologies (53).

The same review emphasized that digital literacy, self-efficacy, social support, and caregiver involvement may influence adherence and effective technology use in cognitively vulnerable older adults (53).

From a digital health perspective, TeleRiab4FRA may provide useful information on the feasibility of implementing advanced telerehabilitation technologies in an older and potentially digitally vulnerable population. The platform combines synchronous audio-video interaction with ECG, heart rate, oxygen saturation, blood pressure, movement monitoring, respiratory tools, and remote adaptation of the rehabilitation plan**.** The assessment of usability, adherence, technical issues, and safety events will help determine whether this type of infrastructure can be realistically integrated into rehabilitation pathways for patients who may have functional limitations, reduced confidence with technology, or require caregiver support.

The use of VO₂peak as the primary outcome (11, 35) strengthens the methodological framework of the study, as it provides an objective and clinically meaningful measure of functional capacity in CHF. At the same time, the inclusion of the Italian Frailty Index as a key secondary endpoint may offer insight into whether changes in aerobic capacity are accompanied by changes in frailty status.

This differs from several frailty-oriented telerehabilitation studies that have mainly used physical performance measures as principal outcomes. Tele-ADHF, for example, used the SPPB as its primary outcome (17), whereas the synchronous frailty protocol by Cigarroa et al. focuses on lower-body strength, cardiorespiratory fitness assessed with a step test, balance, functional status, and quality of life (24).

The exploratory assessment of BNP and ANP may also contribute to the development of future research questions on the biological effects of rehabilitation in frail patients with CHF. Although the present study is not powered to establish mechanistic or omics-based conclusions, the integration of selected biomarker data with functional, cognitive, and digital outcomes may generate preliminary hypotheses on biological profiles associated with frailty and responsiveness to telerehabilitation.

From a clinical and healthcare-system perspective, synchronous telerehabilitation may expand access for patients who face mobility, transportation, or geographical barriers and may allow earlier identification of exercise intolerance or clinical instability. If effective, the model could support continuity of cardiac rehabilitation and more efficient use of specialist resources. Nevertheless, the present protocol is not designed to demonstrate reductions in hospitalization or healthcare costs; these outcomes should be evaluated in future adequately powered implementation and cost-effectiveness studies. Translation into routine care will also depend on digital literacy, access to devices and reliable connectivity, caregiver availability, staff training, interoperability with existing clinical information systems, reimbursement models, and organizational readiness. These factors may generate inequalities in access if implementation is not accompanied by technical support and alternative pathways for digitally vulnerable patients. Future multicenter and implementation studies should therefore assess reach, acceptability, fidelity, equity, cost-effectiveness, and sustainability across different healthcare settings.

If the expected findings are confirmed, TeleRiab4FRA may support the development of scalable and patient-centered telerehabilitation pathways for frail and pre-frail patients with CHF. The study may also provide a methodological basis for larger multicenter trials evaluating the clinical effectiveness, implementation, sustainability, and potential biological correlates of technology-enabled rehabilitation models in real-world healthcare settings.

## 5 Limitations

TeleRiab4FRA has several protocol-level limitations. Its monocentric design and use of a specific technological platform may limit generalizability to centers with different populations, staffing models, or digital infrastructures. In addition, participants and treating professionals cannot be blinded, which may influence subjective outcomes; this risk is partly mitigated by the objective primary endpoint of CPET-derived VO₂peak and by analysis of coded datasets.

Eligibility and retention may be influenced by digital literacy, home connectivity, and caregiver availability, potentially selecting participants who are more able to engage with technology. The active control improves comparability of rehabilitation content but does not permit a direct comparison with center-based cardiac rehabilitation or usual care without structured exercise.

Finally, the 24-week follow-up is sufficient to examine short- to medium-term maintenance but not long-term clinical events, and the trial is powered for VO₂peak rather than for all secondary, caregiver-related, usability, or biomarker outcomes. These outcomes will therefore be interpreted as secondary or exploratory, as prespecified.

## 6 Conclusions and future perspectives

TeleRiab4FRA will test whether a synchronous, sensor-enabled, multidimensional telerehabilitation model provides additional benefit over a content-matched caregiver-supervised home program in frail and pre-frail older adults with CHF. By integrating objective functional assessment, frailty-guided personalization, cognitive and motor training, physiological monitoring, caregiver support, and intervention-fidelity measures, the study is designed to generate clinically and implementation-relevant evidence for a population that is often underrepresented in cardiac rehabilitation research. Future multicenter studies should examine external validity, cost-effectiveness, longer-term clinical outcomes, and integration of this model into routine cardiac and geriatric care pathways. Its potential translation will require subsequent evaluation of cost-effectiveness, digital equity, organizational feasibility, interoperability, and long-term clinical outcomes.

## Data Availability

The original contributions presented in the study are included in the article/Supplementary Material, further inquiries can be directed to the corresponding author.
